# 

**Published:** 1889-05

**Authors:** 


					﻿NOTICE.
A.U articles for publication, books for review, business communications, etc.,
must be sent to Editors, 1123 Spruce Street, Philadelphia.
Subscribers will please notice that this journal will be sent until arrears
are paid- and it is ordered discontinued.
Delinquent Subscribers will please take notice that we
cannot continue to furnish them with this journal unless
they pay up their indebtedness. The Repertory supple-
ment is much too costly to us, and valuable to the profes-
sion to be given away. We have therefore resolved to
stop the service of the journal to all who have not paid up
by the first of July.
We respectfully request that the amount of subscription
be remitted in postal note, money order or draft, and not
in ordinary checks. Money orders, etc., should be made
payable to THE HOMCEOPATHIC PHYSICIAN, and not
to either of the editors.
THE HOMCEOPATHIC PHYSICIAN.
				

